# Assessment of manual adjustment performed in clinical practice following deep learning contouring for head and neck organs at risk in radiotherapy

**DOI:** 10.1016/j.phro.2020.10.001

**Published:** 2020-10-14

**Authors:** Charlotte L. Brouwer, Djamal Boukerroui, Jorge Oliveira, Padraig Looney, Roel J.H.M. Steenbakkers, Johannes A. Langendijk, Stefan Both, Mark J. Gooding

**Affiliations:** a University of Groningen, University Medical Center Groningen, Department of Radiation Oncology, Groningen, The Netherlands; b Mirada Medical Ltd., Oxford, United Kingdom

**Keywords:** Automatic segmentation, Auto-contouring, Deep learning, Contour adjustment, Head and neck organs at risk, Radiotherapy

## Abstract

**Background and purpose:**

Auto-contouring performance has been widely studied in development and commissioning studies in radiotherapy, and its impact on clinical workflow assessed in that context. This study aimed to evaluate the manual adjustment of auto-contouring in routine clinical practice and to identify improvements regarding the auto-contouring model and clinical user interaction, to improve the efficiency of auto-contouring.

**Materials and methods:**

A total of 103 clinical head and neck cancer cases, contoured using a commercial deep-learning contouring system and subsequently checked and edited for clinical use were retrospectively taken from clinical data over a twelve-month period (April 2019–April 2020). The amount of adjustment performed was calculated, and all cases were registered to a common reference frame for assessment purposes. The median, 10th and 90th percentile of adjustment were calculated and displayed using 3D renderings of structures to visually assess systematic and random adjustment. Results were also compared to inter-observer variation reported previously. Assessment was performed for both the whole structures and for regional sub-structures, and according to the radiation therapy technologist (RTT) who edited the contour.

**Results:**

The median amount of adjustment was low for all structures (<2 mm), although large local adjustment was observed for some structures. The median was systematically greater or equal to zero, indicating that the auto-contouring tends to under-segment the desired contour.

**Conclusion:**

Auto-contouring performance assessment in routine clinical practice has identified systematic improvements required technically, but also highlighted the need for continued RTT training to ensure adherence to guidelines.

## 1 Introduction

Deep learning-based auto-contouring for radiation oncology has become an active research field over the past 5 years [1], [2], with both quantitative measures and editing-time suggesting significant improvement over atlas-based auto-contouring [3], [4]. This transition of methods from atlas-based to deep-learning can be traced through the segmentation challenges in radiation oncology; In 2015, all methods entered used atlas- or model-based segmentation approaches [5]. Whereas a split between these methods and deep-learning methods was observed two years later [6], with deep-learning outperforming traditional methods. By 2019, entrants used deep-learning exclusively [7].

Although the majority of commercially available clinical tools still use atlas-based contouring [8], [9], a number of vendors are now offering deep-learning contouring (DLC) [10], [11]. A first step in the process to clinical deployment is validation and commissioning by institutions [12], [13], and a number of validation experiments have been published to support clinical implementation [3], [4]. After the commissioning and the clinical implementation phase, the quality assurance (QA) phase, i.e. ‘model monitoring’ or ‘post-market surveillance’, is needed [13]. This includes the monitoring of user interactions in routine clinical use to determine the true performance impact of the model.

The amount and type of manual adjustments could reveal which structures are generally accepted by clinical experts, if systematic bias exists in DLC output, or if there are patterns of higher variability in adjustments, possibly pointing to higher interobserver variability.

In this study, the user interactions with a commercial DLC system were assessed retrospectively following 12 months of routine clinical use. The main objectives were to identify what manual adjustments were made during clinical review of the automatically generated contours and to identify changes that could be implemented to maximise clinical workflow efficiency. Changes could be related to DLC to reduce systematic errors and improve on robustness in clinical practice, or to training of medical staff members to adhere to contouring guidelines.

## 2 Materials and methods

### 2.1 Patient cohort and contouring

Since March 2019, a head and neck DLC model [4] has been used to support the contouring workflow in the Department of Radiation Oncology of the University Medical Center Groningen. In this workflow for defining organs at risk (OARs), CT scans of head and neck cancer (HNC) patients are automatically transferred to Mirada WorkflowBox 2.0 (Mirada Medical Ltd, Oxford, UK) based on CT protocol name. These are then automatically processed using the DLC model and sent as a DICOM RT Structure Set (RTSS) file to the treatment planning system (RayStation 9A, Raysearch Laboratories, Stockholm, Sweden). A radiation therapy technologist (RTT) checks and adjusts the DLC structures (OARs) following consensus delineation guidelines [14] before treatment planning. The DLC model had previously been trained using 589 HNC cases delineated according to the same guidelines and treated with a range of treatment modalities. Further details on the DLC model training cohort can be found in van Dijk et al. [4] and additional technical details on the deep learning approach can be found in Yang et al. [6].

A total of 103 HNC patients, that went through this clinical workflow in the period April 2019–April 2020, were included in this study. All patients provided informed consent to use their data for research purposes. CT scans were acquired using a large bore 64-slice CT scanner (Somatom AS Open 64-RT Pro, Siemens Medical Systems, Erlangen, Germany), with a 2 mm slice thickness and an in-plane resolution of 1 mm. For the 103 HNC patients, both the auto-generated (DL-contour) and the adjusted structures that had been checked, and possibly edited, by the RTT and used for actual treatment (adjusted contours), were collected. OARs included were carotid arteries, arytenoids, brainstem, buccal mucosa, cerebellum, cerebrum, cricopharyngeal inlet, cervical esophagus, glottic area, mandible, extended oral cavity, parotid glands, pharyngeal constrictor muscles (PCM), spinal cord, submandibular glands, supraglottic larynx and thyroid gland. Most contours (95 patients) were checked and edited by a member of the dedicated RTT HNC contouring team (observer A (36), B (34) and C (25)).

In some patients, certain DL-contours were missing since no output was predicted (mostly the arytenoid). Also, a structure could be missing (surgically removed), infiltrated by the tumour or insignificant for a specific patient and, therefore, the contour was deleted by the RTT and thus not present as an adjusted contour. The number of available DL- and adjusted contour pairs therefore varied with a median of 95 (range 38–103). Numbers per structure can be found in the Supplementary Tables.

### 2.2 Evaluation of contour editing

Previously conducted interobserver studies required multiple observers contour all the OARs on the same CT image [15], [16]. However, in this study different patients are being considered. Therefore, it was necessary to align all contours of a given organ to a common reference to evaluate the anatomical location of adjustments over the population of patients. A reference shape could be constructed per OAR, defined as the statistical mean shape of the OAR over all patients [17]. However, such a mean shape may not have a realistic anatomy, and therefore, a more representative shape was acquired for meaningful interpretation of results. For this purpose, the shape with the median volume over all patients on an organ-by-organ basis was selected out of the adjusted contours followed by a visual check by a clinical expert (RS). For the extended oral cavity, topological differences were noted between the observed surfaces due to different closure of the mouth or position of the tongue and uvula. Therefore, four reference shapes, denoted references I–IV, were selected that reflected the main categories of observed topologies. For the glottic area, two reference shapes were required to represent the two main observed topologies, denoted reference I and II.

First, a 3D continuous surface was reconstructed from the RTSS representation of the adjusted contours. The continuous surface was then sampled at the CT voxel resolution and converted to a 3D discrete mesh representation. The degree of manual adjustment performed on the DL-contour by a clinical expert prior to treatment planning was calculated for each of these mesh vertices. The degree of adjustment was taken to be the shortest surface displacement from the edited contour to the corresponding DL-contour. Conversion to a 3D mesh representation from the 2D contour-based RTSS representation, as output by the clinical software, allows this adjustment to be measured and recorded on the superior/inferior surfaces of structures, and also enables 3D registration of the structures to be performed. The uniform sampling at CT resolution was used as this was deemed the precision to which the image can represent the anatomy, while providing a dense surface representation.

To analyse where and to what extend the DL-contours were adjusted over the population, it was necessary to register all examples to the selected common reference surface for each considered OAR. This was achieved using a deformable shape-to-shape registration technique to enable localized analysis and comparison of the manual adjustments. Since a predefined point-to-point correspondence was not available, a type of Iterative Closest Point (ICP) registration algorithm [18] was used. A comprehensive methodological survey was done by Tam et al. [19] and an overview with a focus on medical imaging application was provided by Audette et al. [20]. Specifically, the approach used was based on the locally affine weighted ICP algorithm described in [21]. An initial rigid transformation was first estimated with a rigid ICP algorithm and used as an initialisation for the non-rigid algorithm. The correct registration between different patients is hard to define, since there may not be a “correct” anatomical correlation to be made. However, gross misregistration may result in adjustments being assigned to an incorrect anatomical location spatially. Therefore, in both algorithms, a weighting factor, based on the similarity of the meshes’ normals, was applied to the distance-matching cost function of the registration optimisation to improve robustness to mismatched points.

For every vertex point the median (50th percentile) and range (10th–90th percentiles) of the adjustments were calculated over all patients for the analysis. Percentiles were used as the distribution of adjustments cannot be assumed to be symmetric and these measures are more robust to outliers. A low median adjustment and low range could be interpreted as general acceptance of DL-contours, while high median adjustment and low range indicates a systematic error in the DL-contour, which may need correction by improving the auto-contouring system. Low median adjustment and high range may indicate that DL-contouring performed well in general but there may be situations where it failed, or that there is a region consisting of high interobserver variability in adjustment.

To report on the amount of adjustment for a particular substructure, the reference shapes were segmented in anatomical sub-regions as described by Brouwer et al. [16], and the degree of adjustment in each region was calculated.

## 3 Results

**Fig. 1:**
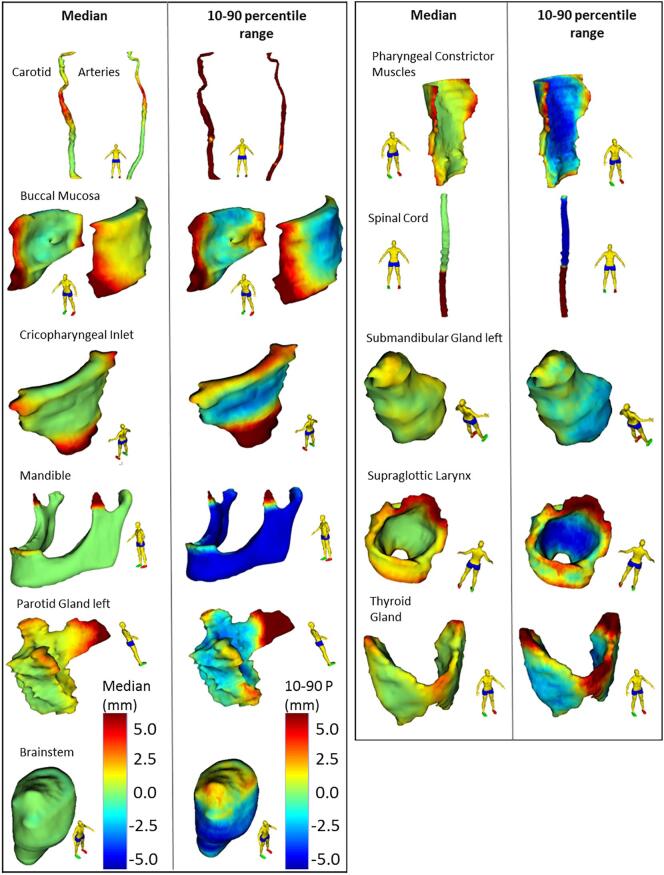
Median and 10–90 percentile range Adjustments projected on the reference shape of each organ at risk (OAR). Positive Adjustments reflect an outward correction of the DL-contour. The 10–90 percentile range represents the variability in Adjustment between patients. The Cerebellum, Cerebrum, Arytenoids and Cervical Esophagus were not displayed, Adjustments for these OARs were small or evenly distributed over the reference structure.

**Fig. 2:**
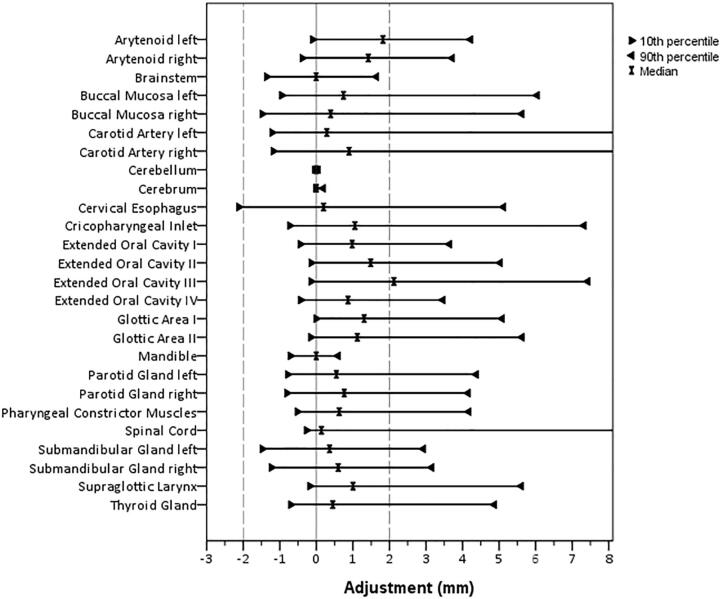
Median, 10, and 90 percentile of Adjustments over all patients per organ at risk. For Spinal Cord and Carotid Artery Left and Right, the 90 percentile was cut from the axis at 72, 45 and 40 mm, respectively.

**Fig. 3:**
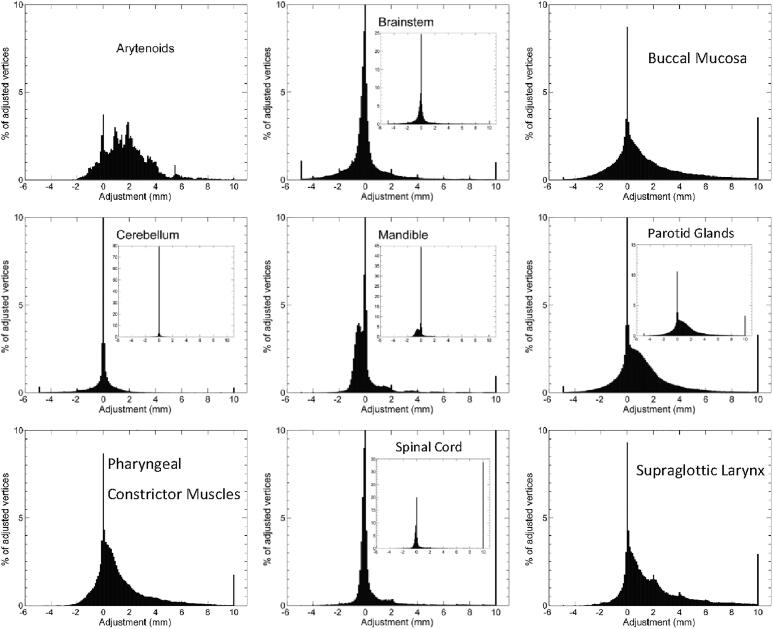
Histograms of Adjustments over all patients for Arytenoid, Brainstem, Buccal Mucosa, Cerebellum, Mandible, Parotid Glands, Pharyngeal Constrictor Muscles, Spinal Cord and Supraglottic Larynx.

For the majority of structure vertex points, the median value of editing was low, i.e. <2 mm (Fig. 1). Also, the median of adjustments per structure was within 2 mm (Fig. 2) for all structures. Median adjustments were all positive, indicating the overall editing was enlarging the DL-contours. The distribution of adjustments was asymmetric for most structures (Figs. 2 and 3). For example, the supraglottic larynx presents a very skew distribution of (larger) adjustments. Also, maxima in the histogram can be observed every 2 mm, corresponding to the CT slice thickness and editing of structures in cranio-caudal direction. For the arytenoid, many adjustments were done between 2 and 4 mm, and for the brainstem almost no adjustments were done, except for 1% of 10 mm adjustments (Fig. 3). For the spinal cord, > 33% of vertices were adjusted ≥10 mm (Fig. 3), which corresponds to the caudal end (Fig. 1).

**Fig. 4:**
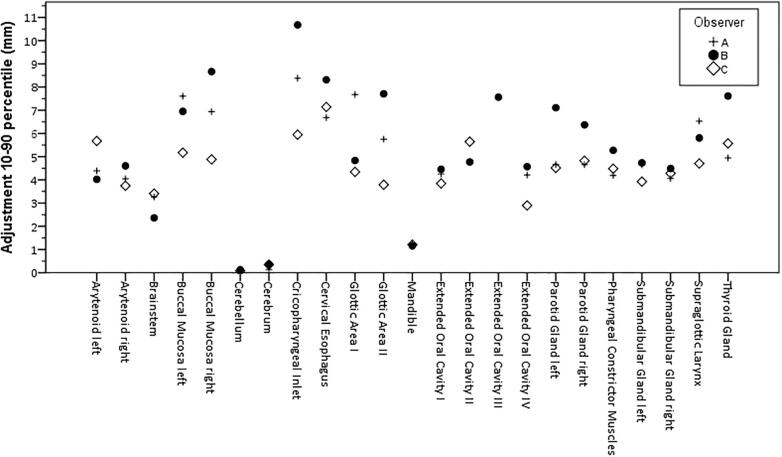
The 10–90 percentile Adjustment range per structure for Observer A, B and C.

Median adjustments for observer A, B and C were all within 2 mm. For observer B, the range of adjustments was slightly higher than for A and C (Fig. 4).

Regarding the sub regions, systematic (median) adjustments >2 mm were identified for the cranial part of the arytenoid, anterior part of the buccal mucosa, caudal part of the cricopharyngeal inlet, cranial part of the glottic area, anterior part of the PCM, caudal part of the spinal cord and the caudal part of the supraglottic larynx (Supplementary Figs. 1 and 2 and Supplementary Table 1).

The largest 10–90 percentile range was found for the carotid arteries. Adjustments were up to several centimetres for the cranial subregion (Supplementary Table 2). Visual inspection of outlier cases indicated that the largest contour adjustments were done in case of larger (nearby or surrounding) tumours and in case of severe dental artefacts. Also, parotid glands with larger adjustments contained larger tissue heterogeneity.

The largest median of adjustments was found for oral cavity reference topology III representing elongated, open mouth (2.1 mm global, 2.9 mm caudal) (Supplementary Table 3 and Supplementary Fig. 2). DL-contours for oral cavity in case of a closed mouth (reference I and IV) needed less adjustments (median ≤ 1 mm), with largest variation in adjustment for the caudal subregion (10–90 percentile 6.8 and 4.5 mm, respectively (Supplementary Tables 3 and 4). The glottic area topologies did not result in different findings, the cranial subregion was edited most with median adjustments of 2.6 and 2.1 mm and a 10–90 percentile range of 8.3 and 8.6 mm for reference I and II, respectively (Supplementary Tables 3 and 4).

## 4 Discussion

This assessment of manual adjustment of deep learning contouring in clinical practice identified small systematic editing overall (median of editing < 2 mm). Still, regions of structures with higher systematic editing were identified and for some organs large variability in editing (large 10–90 percentile range of manual adjustments) occurred in this patient cohort.

The variability in editing was low for the cerebrum, cerebellum, brainstem and mandible (within 3 mm), suggesting these contours are generally accepted without the need for editing. For the other OARs, the variability in adjustments (10–90 percentile range) was larger. The region of high systematic editing in the coronoid process of the mandible suggested an artefact in the auto-contouring system that was removing small disconnected 2D regions for this structure. This has since been rectified as a result of this study.

A number of identified adjustments can be explained by variation in interpretation of guidelines by the RTT. For the spinal cord, manual adjustments to the caudal border were found up to 80 mm and are clearly visible in Fig. 1. This can be explained in two ways; First, the RTTs did not consistently comply with the delineation guidelines which denotes the caudal border as the upper edge of T3, but instead delineated the spinal cord for the entire length of the CT scan. Second, the guideline recommends increasing the caudal border up to at least 5 cm caudal to the PTV, which results in a variable caudal border for each patient. DLC was trained on a set of clinical data, where the majority of cases would stop at T3. Although it was expected that DLC would stop contouring at this point, it was also expected that manual adjustment would occur here depending on the PTV extent. DLC might be improved by contouring the whole spinal cord, as it is generally easier to delete than add [22]. However, such analysis would still demonstrate adjustment is required here as the guidelines accept some variation in where to stop. Despite needing correction caudally, no editing was required for the remaining spinal cord, resulting in a very low overall median adjustment. The identification of inconsistencies to delineation guidelines was also presented by Men et al. [23], were DLC is presented as a tool for QA of clinical trial data.

For the PCM, the anterior adjustments can be explained by the fact this structure is often more extended anteriorly than described in the delineation guideline. A discrepancy between the training dataset – which was contoured consistently according to the guideline – and delineation in daily practice may explain this editing pattern. This would indicate a need to improve training RTT for compliance to guidelines. Alternatively, an update to guidelines may be required.

The buccal mucosa was edited with the largest variability anteriorly, as in a number of cases, the lips were erroneously included in the adjusted contour as well. This inconsistent adjustment could be reduced by including the lips to the clinical OAR set (which is currently not the case) and subsequently to the DLC model.

The parotid glands showed variability in adjustments for the anterior part (Fig. 1, Fig. 3). The anterior extension of the parotid gland along the main parotid duct varies between patients, and is prone to high interobserver variability [16]. For the cranial part of the parotid gland, systematic editing was observed to enlarge the structure in cranial direction, while the 10–90 percentile range was lower (Fig. 1). To improve this, DLC would need further training to increase the cranial extent of the parotid gland. Generally, DLC seems to under-segment. DLC is trained on a broad range of data subject to inter-observer variability, which potentially results in an averaging effect and smoothing of the contours which naturally shrinks them.

For the supraglottic larynx, a similar effect as for the parotid was observed; for the cranial region, both median and range were large, suggesting a variable amount of enlarging this structure. This suggests high editing variability but in a consistent direction. It implies DLC is inaccurate in a proportion of cases. To discriminate relevant manual adjustments from interobserver variability, additional research is needed including multiple observers adjusting the same set of patients. High interobserver variability was observed for the glottic larynx previously [16], particularly in caudal, medial and posterior regions.

Since many adjustments were done within 2 mm, the question arises to what degree contour adjustments are clinically meaningful. As other studies have shown, variability in contouring does not result in significant dosimetric differences for the majority of organs [4], [24]. A threshold for meaningful contour adjustments could possibly be set [22], and RTTs trained to accept contours in cases not exceeding this threshold. Practical tools to guide the user in this decision-making process will enable further automation of contouring practice. Further research could also consider the dosimetric impacts of the edits made within this cohort.

While a slightly larger range of adjustments was observed for observer B (Fig. 4), primarily for the salivary glands and swallowing structures, the overall median and regional amount of editing was similar over observers suggesting no systematic differences between observers. No demographic or clinical meta-data balancing was performed when collecting clinical patient data for this study, thus no strong conclusions on differences between observers can be made as it is uncertain that the patient populations are equivalent for each observer.

Finally, a number of differences in current clinical practice compared to the DLC training set could have influenced the amount of editing. The training set of the DLC model contained patient scans of a different immobilization system and couches, less hyperextended than the current clinical system. Variability in the amount of manual adjustment could also be explained by different tumour locations, the volume of the tumour, compromised CT quality or abnormal OAR anatomy. Follow-up assessments could investigate manual adjustments of subgroups of larger patient numbers to identify more advanced strategies of improving contouring practice.

Overall, the evaluation of manual adjustments to auto-contours performed in routine clinical practice is a valuable tool for identifying ways to improve quality and efficiency of contouring practice, enabling demonstration of areas for improvement of auto-contouring, the need for training of those adjusting the contours on the guidelines, and potentially a lack of clarity or uncertainty in guidelines.

## Declaration of Competing Interest

The authors declare that they have no known competing financial interests or personal relationships that could have appeared to influence the work reported in this paper.
