# Supplementary material for: Assessment of manual adjustment performed in clinical practice following deep learning contouring for head and neck organs at risk in radiotherapy
Source: Phys Imaging Radiat Oncol. 2020 Oct 14;16:54–60. doi: 10.1016/j.phro.2020.10.001 (PMC7807591; doi:10.1016/j.phro.2020.10.001)

**Supplementary Table 1.** Median Adjustments (mm) for all structures. overall (global) and per sub region. N= number of structures included. Adjustments > 2 mm displayed in bold.

|  | N | **Global** | **Subregion** | | | | | | |
| --- | --- | --- | --- | --- | --- | --- | --- | --- | --- |
|  |  |  | Cranial | Caudal | Anterior | Posterior | Medial | Lateral |  |
| Carotid Artery left | 71 | 0.3 | 1.0 | 0.0 | . | . | 0.4 | . |  |
| Carotid Artery right | 71 | 0.9 | 0.8 | 0.3 | . | . | 1.8 | . |  |
| Arytenoid left | 40 | 1.8 | **2.1** | 2.0 | 2.0 | 1.4 | . | . |  |
| Arytenoid right | 38 | 1.4 | **2.1** | 2.0 | 1.3 | 1.1 | . | . |  |
| Brainstem | 103 | 0.0 | 0.0 | 0.0 | 0.0 | 0.0 | . | . |  |
| Buccal Mucosa left | 95 | 0.7 | 0.5 | 2.0 | **3.4** | 0.5 | 0.2 | 0.9 |  |
| Buccal Mucosa right | 97 | 0.4 | 1.2 | 0.8 | **3.6** | 0.4 | 0.0 | 0.4 |  |
| Cerebellum | 98 | 0.0 | 0.0 | 0.0 | 0.0 | 0.0 | . | 0.0 |  |
| Cerebrum | 98 | 0.0 | 0.0 | 0.0 | 0.0 | 0.0 | 0.0 | 0.0 |  |
| Cricopharyngeal Inlet | 95 | 1.1 | 1.3 | **5.4** | . | . | 1.0 | 0.5 |  |
| Cervical Esophagus | 95 | 0.2 | 0.1 | 0.0 | . | . | -0.3 | 0.4 |  |
| Glottic Area | 65 | 1.1 | **2.2** | 1.8 | - | 0.8 | 1.1 | 1.4 |  |
| Mandible | 100 | 0.0 | 0.2 | 0.0 | 0.0 | 0.0 | . | . |  |
| Extended Oral Cavity | 72 | 1.1 | 0.8 | 0.8 | 1.2 | 1.1 | - | - |  |
| Parotid Gland left | 99 | 0.6 | 1.5 | 0.5 | 1.2 | 0.1 | 0.6 | 0.0 |  |
| Parotid Gland right | 102 | 0.8 | 1.3 | 0.3 | 0.9 | 0.4 | 0.8 | 0.6 |  |
| Pharyngeal Constrictor Muscles | 71 | 0.6 | 1.4 | 2.1 | **2.7** | . | 0.5 | 0.5 |  |
| Spinal Cord | 101 | 0.1 | 0.0 | **3.6** | . | . | 0.0 | . |  |
| Submandibular Gland left | 92 | 0.4 | 0.8 | 0.4 | 0.8 | 0.1 | 0.0 | 0.5 |  |
| Submandibular Gland right | 90 | 0.6 | 1.2 | 0.6 | 0.5 | 0.7 | 0.0 | 0.8 |  |
| Supraglottic Larynx | 96 | 1.0 | 1.7 | **2.1** | 0.9 | 1.4 | 0.4 | . |  |
| Thyroid Gland | 98 | 0.5 | 0.7 | 0.6 | . | 0.1 | 1.0 | 0.2 |  |

**Supplementary Table 2**. The 10-90 percentile Adjustments (mm) for all structures. Overall (global) and per subregion. N= number of structures included. Range > 5 mm displayed in bold.

|  | N | **Global** | **Subregion** | | | | | |
| --- | --- | --- | --- | --- | --- | --- | --- | --- |
|  |  |  | Cranial | Caudal | Anterior | Posterior | Medial | Lateral |
| Carotid Artery left | 71 | **46.4** | **86.9** | **22.1** | **.** | **.** | **31.6** | . |
| Carotid Artery right | 71 | **40.7** | **89.3** | **24.9** | **.** | **.** | **37.2** | . |
| Arytenoid left | 40 | 4.3 | **5.9** | 4.1 | 4.0 | 4.3 | . | . |
| Arytenoid right | 38 | 4.1 | **5.4** | 4.4 | 3.7 | 3.6 | . | . |
| Brainstem | 103 | 3.0 | **5.7** | 3.9 | 1.6 | 1.6 | . | . |
| Buccal Mucosa left | 95 | **7.0** | **5.8** | **9.0** | **11.2** | **5.4** | 4.9 | **5.5** |
| Buccal Mucosa right | 97 | **7.1** | **8.6** | **6.3** | **10.8** | **6.2** | **5.5** | 5.0 |
| Cerebellum | 98 | 0.1 | 0.1 | 0.1 | 0.2 | 0.1 | . | 0.1 |
| Cerebrum | 98 | 0.2 | 0.1 | 0.9 | 0.2 | 0.1 | 0.9 | 0.2 |
| Cricopharyngeal Inlet | 95 | **8.0** | **7.6** | **15.3** | **.** | **.** | **5.5** | **6.1** |
| Cervical Esophagus | 95 | **7.2** | **7.8** | **8.6** | **.** | **.** | **6.9** | **5.6** |
| Glottic Area | 65 | **5.6** | **8.5** | 4.7 | - | 3.2 | **6.4** | 4.9 |
| Mandible | 100 | 1.3 | **9.6** | 0.8 | 2.9 | 0.8 | . | . |
| Extended Oral Cavity | 72 | 4.3 | 2.9 | **6.1** | 4.2 | 4.4 | - | - |
| Parotid Gland left | 99 | **5.2** | **5.6** | **5.6** | **8.9** | 3.6 | 4.3 | 2.0 |
| Parotid Gland right | 102 | 5.0 | **5.2** | **5.8** | **7.5** | 4.3 | 4.5 | 2.6 |
| Pharyngeal Constrictor Muscles | 71 | 4.7 | **6.3** | **9.2** | **7.4** | . | 3.4 | 4.0 |
| Spinal Cord | 101 | **72.0** | 2.3 | **79.9** | . | . | 0.6 | . |
| Submandibular Gland left | 92 | 4.4 | **5.4** | 3.9 | 3.8 | 5.0 | 4.1 | 4.3 |
| Submandibular Gland right | 90 | 4.4 | **5.1** | 3.5 | 3.7 | 5.0 | 4.2 | 4.0 |
| Supraglottic Larynx | 96 | **5.8** | **8.1** | **6.6** | 4.1 | **6.4** | 3.4 | . |
| Thyroid Gland | 98 | **5.6** | **7.4** | 4.5 | . | 3.0 | **7.9** | 3.9 |

**Supplementary Table 3.** Median Adjustments (mm) for (selected topologies of) Glottic Area and Extended Oral Cavity. Overall (global) and per subregion. N= number of structures included.

|  | N | **Global** | **Subregion** | | | | | |
| --- | --- | --- | --- | --- | --- | --- | --- | --- |
|  |  |  | Cranial | Caudal | Anterior | Posterior | Medial | Lateral |
| Glottic Area I | 14 | 1.3 | 2.6 | 2 | - | 0.8 | 1.8 | 1.4 |
| Glottic Area II | 51 | 1.1 | 2.1 | 1.7 | - | 0.8 | 0.9 | 1.4 |
| Glottic Area | 65 | 1.1 | 2.2 | 1.8 | - | 0.8 | 1.1 | 1.4 |
| Extended Oral Cavity I | 41 | 1 | 0.6 | 0.6 | 1.1 | 1.1 | . | . |
| Extended Oral Cavity II | 4 | 1.5 | 1.6 | 1 | 1.7 | 1.4 | . | . |
| Extended Oral Cavity III | 4 | 2.1 | 2.4 | 2.9 | 2 | 2.2 | . | . |
| Extended Oral Cavity IV | 23 | 0.9 | 0.7 | 0.6 | 1.1 | 0.9 | . | . |
| Extended Oral Cavity | 72 | 1.1 | 0.8 | 0.8 | 1.2 | 1.1 | - | - |

**Supplementary Table 4.** Range (10-90%) (mm) for (selected topologies of) Glottic Area and Extended Oral Cavity. Overall (global) and per subregion. N= number of structures included.

|  | N | **Global** | **Subregion** | | | | | |
| --- | --- | --- | --- | --- | --- | --- | --- | --- |
|  |  |  | Cranial | Caudal | Anterior | Posterior | Medial | Lateral |
| Glottic Area I | 14 | 5.1 | 8.3 | 4.2 | . | 2.7 | 6.9 | 4.1 |
| Glottic Area II | 51 | 5.8 | 8.6 | 4.9 | . | 3.4 | 6.2 | 5.1 |
| Glottic Area | 65 | 5.6 | 8.5 | 4.7 |  | 3.2 | 6.4 | 4.9 |
| Extended Oral Cavity I | 41 | 4.1 | 2.2 | 6.8 | 4.0 | 4.1 | . | . |
| Extended Oral Cavity II | 4 | 5.2 | 8.0 | 4.9 | 4.6 | 5.8 | . | . |
| Extended Oral Cavity III | 4 | 7.6 | 9.1 | 9.9 | 6.8 | 8.0 | . | . |
| Extended Oral Cavity IV | 23 | 3.9 | 2.1 | 4.5 | 4.1 | 4.0 | . | . |
| Extended Oral Cavity | 72 | 4.3 | 2.9 | 6.1 | 4.2 | 4.4 |  |  |

**Supplementary Figure 1**. Median, 10, and 90 percentile Adjustments over all patients per subregion of the organ at risk. The 90th percentile was cut from the axis for Carotid Artery left (86.9 mm cranial, 22.1 mm caudal, 31.6 mm medial), Carotid Artery right (89 mm cranial, 25 mm caudal, 37 mm medial), and Spinal Cord (80 mm caudal)

**
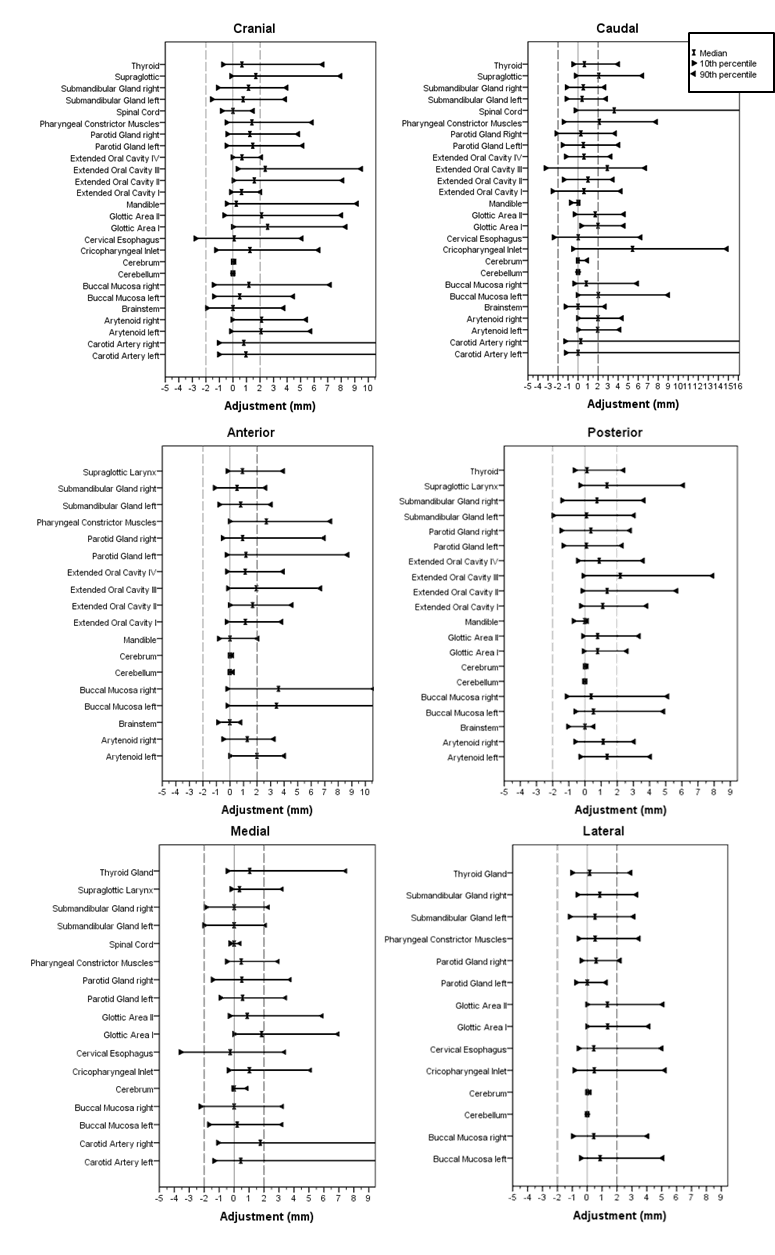
**

**Supplementary Figure 2**. Median and 10-90 percentile range Adjustment projected on the reference shape for the Oral Cavity and Glottic Area reference topologies.


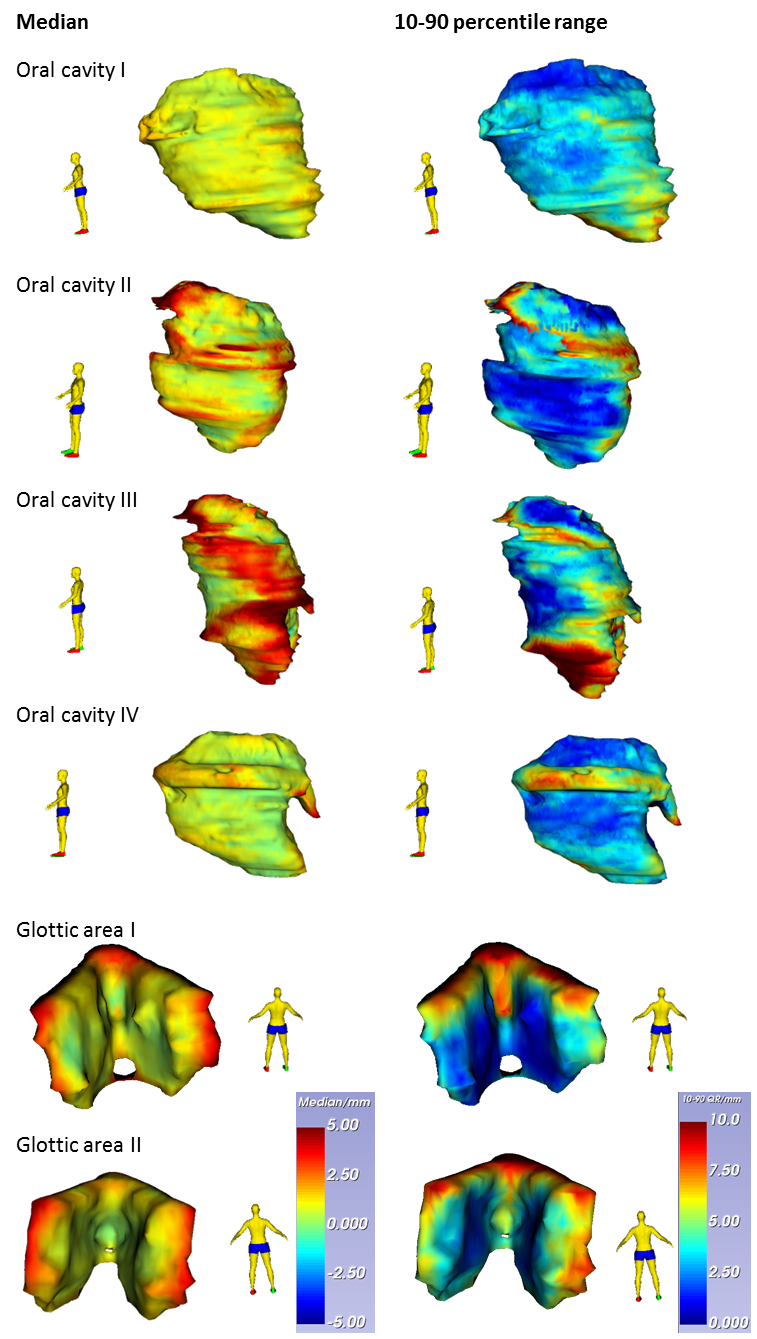

Supplement: Supplementary data 1 [file mmc1.docx]
